# Analysis of risk factors and prevention strategies for functional delayed gastric emptying in 1243 patients with distal gastric cancer

**DOI:** 10.1186/s12957-020-02085-2

**Published:** 2020-11-19

**Authors:** Tao Pang, Xiao-Yi Yin, Hang-Tian Cui, Zheng-Mao Lu, Ming-Ming Nie, Kai Yin, Guo-En Fang, Tian-Hang Luo, Xu-Chao Xue

**Affiliations:** 1 Department of Gastrointestinal Surgery, Changhai Hospital, The Second Military Medical University, 168 Changhai Road, Shanghai, 200433 China; 2 Department of Hepatobiliary Pancreatic Surgery, Changhai Hospital, The Second Military Medical University, Shanghai, 200433 China

**Keywords:** Gastric cancer, Functional delayed gastric emptying, Gastrointestinal decompression

## Abstract

**Background:**

Analysis of the risk factors associated with functional delayed gastric emptying after distal gastric cancer surgery to provide a basis for further reduction of the incidence of this complication.

**Methods:**

Total of 1382 patients with distal gastric cancer from January 2016 to October 2018 were enrolled. Correlation analysis was performed in 53 patients with FDGE by logistic regression. Subgroup risk analysis was performed in 114 patients with preoperative pyloric obstruction. A Pearson Chi-square analysis was used to compare categorical variables between normal distribution groups. Meanwhile, a *t* test was used to compare continuous variables between groups. Odds ratio (OR) was used for comparison of the two groups, and it was summarized with its 95% confidence interval (CI) and *p* value using logistic regression.

**Result:**

In multivariable analysis, age (OR 1.081, 95% CI, 1.047–1.117), BMI (OR 1.233, 95% CI, 1.116–1.363), preoperative pyloric obstruction (OR 3.831, 95% CI, 1.829–8.023), smaller volume of residual stomach (OR 1.838, 95% CI, 1.325–6.080), and anastomosis in greater curvature perpendicular (OR 3.385, 95% CI, 1.632–7.019) and in greater curvature parallel (OR 2.375, 95% CI, 0.963–5.861) were independent risk factors of FDGE. In the preoperative pyloric obstruction group, higher BMI (OR 1.309, 95% CI, 1.086–1.579) and preoperative obstruction time (OR 1.054, 95% CI, 1.003–1.108) were independent risk factors of FDGE and preoperative gastrointestinal decompression (OR 0.231, 95% CI, 0.068–0.785) was independent protective factor of FDGE.

**Conclusion:**

Adequate gastrointestinal decompression should be performed before the operation to reduce the incidence of postoperative gastroparesis in patients with preoperative pyloric obstruction. We also could improve the surgical methods to reduce the occurrence of FDGE, such as controlling the size of the residual stomach, ensuring blood supply. Especially selecting an appropriate stapler and anastomosis during the anastomosis process, the occurrence of FDGE can be reduced.

**Supplementary Information:**

The online version contains supplementary material available at 10.1186/s12957-020-02085-2.

## Background

Gastric cancer (GC) is the fifth most common cancer, ranking third as a cause of cancer-related death globally [1]. GC patients are often asymptomatic in early stage; therefore, most patients are diagnosed at an advanced stage. Adequate surgical resection is the only curative therapeutic option for most GC [2], while endoscopic procedures are recommended in low probability lymph node metastasis cases, and when lesion size and site are suitable for whole resection [3]. The extent of surgery is determined by tumor location, diameter, and histological type. Patients with GC in the lower two-thirds of the stomach can often be treated with subtotal or distal gastrectomy, which often leads to post-gastrectomy syndromes such as functional delayed gastric emptying (FDGE), dumping syndrome, reduced food intake, and reflux esophagitis [4]. FDGE, which is a common complication after distal gastrectomy, has an incidence of 10% to 15% of which 5% to 10% with clinical symptoms. This complication not only causes patients to suffer from eating but also leads to the reduction of the patients’ quality of life, and the increase of hospital stays and medical expenses, and the increase the workload and psychological pressure on doctors [5, 6]. Most patients with FDGE recover after conservative treatment within 1–2 months, while few of them might need a longer time or even another operation. And there are yet to be put forward effective cure methods. Although several studies have focused on FDGE, the reason remains unclear.

The aim of this study was to investigate the pathogenic factors associated with surgery or pathology, and to avoid and reduce the occurrence of this complication after gastric cancer surgery.

## Materials and methods

### Patients

We reviewed the records of patients with pathologically diagnosed gastric cancer, in which 1382 patients were treated with radical distal gastrectomy at the Department of General Surgery, Shanghai Changhai Hospital between 1st January 2016 and 1st January 2019. These cases were enrolled in the present study. Patients who underwent combined devisceration, such as vessel or combined adjacent organ resection caused by tumor invasion, who received preoperative chemotherapy or radiotherapy or who were diagnosed with diabetics were excluded. The number of patients excluded by each category was 17, 20, and 12 respectively. Finally, 1243 patients were included in this study.

Among the remaining 1243 patients, 53 patients were diagnosed with postoperative FDGE. All cases were diagnosed according to the following criteria: one or multiple tests suggesting there was no mechanical obstruction in the gastric outflow tract; the drainage volume from the stomach was > 800 ml/day and lasted for more than 10 days; there was no obvious water–electrolyte imbalance; FDGE was not induced by conditions such as diabetes, hypothyroidism, and connective tissue disease; and no drugs affecting contraction of smooth muscle were used. Suspected FDGE cases without upper gastroenterography and gastroscope examination were further excluded in the following analysis (Supplementary Material).

Clinicopathologic features such as age, gender, body mass index (BMI), smoking history, and history of abdominal surgery were reviewed. Preoperative pyloric obstruction, preoperative albumin, preoperative hemoglobin, postoperative albumin, postoperative hemoglobin, and preoperative anxiety were also reviewed.

### Surgical procedures

All patients underwent radical gastrectomy for a primary tumor and D2+ lymph node dissection for cases of advanced gastric cancer and D2 or D1+ lymph node dissection for cases of early gastric cancer by five experienced surgeons, who have performed more than 300 cases of gastrectomy.

Distal gastrectomy was routinely performed. Among the cases, 25-mm circular staplers or 60-mm linear cutters were used for gastrojejunostomies for digestive track reconstruction using both conventional open and laparoscopy assisted gastrectomy. The volume of residual stomach was determined by the location and size of the tumor, and we defined the residual stomach size according to whether the left gastroepiploic artery or the first branch of the short gastric artery were ligated. All cases had R0 resection performed. Hand-sewn anastomoses were performed for Braun anastomosis. End-to-side gastrojejunostomy was made approximately 35 cm distal to the ligament of Treitz via the anterocolic and isoperistaltic pathway in uncut cases. While in Roux-en-Y cases, the jejunum was cut off 40 cm distal to the ligament of Treitz. Braun anastomosis was performed about 6 to 8 cm distal to the gastrojejunostomy, and the uncut procedure was conducted by ligating jejunum with 0 braided silk suture 3 cm distal to the gastrojejunostomy in uncut cases. The gastrojejunostomy anastomosis was located in the posterior wall of gastric body.

### Preoperative pyloric obstruction

Patients who were diagnosed with preoperative pyloric obstruction received CT scanning and gastroscopy, depending on the symptoms like abdominal distention and vomiting. Gastrointestinal decompression can lead to outflow of persistent food. The level of obstruction was classified by the amount of residual food and how long the obstruction happened.

### Statistical analysis

Clinical characteristics of patients were summarized, as well as described specifically for subgroups by descriptive statistics. After descriptive analyses were performed, a Pearson Chi-square analysis was used to compare categorical variables between normal distribution groups. A Fisher’s exact test was used in the abnormal distribution groups. While a *t* test was used to compare continuous variables between groups.

Odds ratio (OR) for comparison of the two groups was summarized with its 95% confidence interval (CI) and *p* value using logistic regression. The multivariate model was created using a backward elimination method, and the probability was set at 0.20 for removal. ORs were also adjusted for factors affecting the response variable. *P* values lower than 0.05 were considered statistically significant. All statistical analyses were carried out using SPSS Statistics ver. 23.0 (IBM Co., Armonk, NY, USA).

## Results

The patients’ clinicopathological characteristics were shown in Table 1. Among the 1243 patients, 801 (64.4%) patients were men and 442 (35.6%) were women with a mean age of 53.444 ± 9.389 years. The average operation time was 186.238 ± 21.486 min, and the average surgical bleeding volume was 171.440 ± 52.732 ml. There were no significant differences in the clinical characteristics between FDGE and none FDGE group, either within the sex, smoking history, history of abdominal surgery, preoperative albumin, preoperative hemoglobin, postoperative albumin, postoperative hemoglobin, preoperative anxiety, operation time, operation blood loss, operation approach, reconstruction methods, and position of afferent loop (*p* > 0.05). However, the mean age was 62.151 ± 11.360 years in FDGE group and 53.056 ± 9.105 years in none FDGE group (*p* < 0.001). The BMI index was 24.032 ± 2.670 kg/m^2^ in FDGE group and 22.078 ± 3.086 kg/m^2^ in none FDGE group (*p* < 0.001). And 27 (50.9%) patients who suffered preoperative pyloric obstruction showed FDGE out of 53 patients, compared with 87 (7.3%) out of 1190 patients (*p* < 0.001). In the aspect of volume of residual stomach, the numbers of LGPA preserved, LGPA ligation and first branch of SGA ligation were 14 (26.4%), 17 (32.1%), and 22 (41.5%) in FDGE group, while the numbers in none FDGE group were 546 (45.9%), 305 (25.6%), and 339 (28.5%) (*p* = 0.018), respectively. As for position of anastomosis, the numbers of greater curvature parallel, greater curvature perpendicular, middle position of gastric posterior wall were 10 (18.9%), 21 (39.6%), and 22 (41.5%) in FDGE group, compared with 132 (11.1%), 201 (16.9%), and 857 (72.0%) in none FDGE group (*p* < 0.001). And 29 (54.7%) patients who were performed by linear stapler showed FDGE (*p* < 0.001).

**Table 1:** General characteristics of 1243 patients

Items	Overall (*n* = 1243)	With FDGE (*n* = 53)	Without FDGE (*n* = 1190)	*P* value
Male sex	801 (64.4%)	35 (66.0%)	766 (64.6%)	0.884
Age (years)	53.444 ± 9.389	62.151 ± 11.360	53.056 ± 9.105	< 0.001
BMI(kg/m^2^)	22.162 ± 3.095	24.032 ± 2.670	22.078 ± 3.086	< 0.001
Smoking history	256 (20.6%)	14 (26.4%)	242 (20.3%)	0.298
History of abdominal surgery	160(12.9%)	7(13.2%)	153(12.9%)	0.836
Preoperative pyloric obstruction	114(9.2%)	27(50.9%)	87(7.3%)	< 0.001
Preoperative albumin (g/L)	42.022 ± 4.344	41.556 ± 4.610	42.042 ± 4.333	0.435
Preoperative hemoglobin (g/L)	137.370 ± 12.745	131.415 ± 23.742	130.324 ± 12.041	0.741
Postoperative albumin (g/L)	34.139 ± 3.731	32.226 ± 4.060	34.135 ± 3.717	0.862
Postoperative hemoglobin (g/L)	115.930 ± 13.409	119.264 ± 22.059	115.782 ± 12.885	0.259
Preoperative anxiety				0.958
None	751 (60.4%)	32 (60.4%)	719 (60.4%)	
Counseling	219 (17.6%)	11 (20.8%)	262 (22.0%)	
Drugs	273 (22.0%)	10 (18.9%)	209 (17.6%)	
Operation time (min)	186.238 ± 21.486	189.094 ± 52.711	186.111 ± 18.982	0.683
Operation blood loss (ml)	171.440 ± 52.732	181.132 ± 89.911	171.008 ± 50.915	0.388
Approach				0.183
Opening	832 (66.9%)	31 (58.5%)	801 (67.3%)	
Laparoscopy	411 (33.1%)	22 (41.5%)	389 (32.7%)	
Reconstruction				0.079
Roux-en-Y	442 (35.6%)	25 (47.2%)	417 (35.0%)	
Uncut Roux-en-Y	801 (64.4%)	28 (52.8%)	773 (65.0%)	
Volume of residual stomach				0.018
LGPA preserved	560 (45.1%)	14 (26.4%)	546 (45.9%)	
LGPA ligation	322 (25.9%)	17 (32.1%)	305 (25.6%)	
First branch of SGA ligation	361 (29.0%)	22 (41.5%)	339 (28.5%)	
Position of anastomosis				< 0.001
Greater curvature parallel	142 (11.4%)	10 (18.9%)	132 (11.1%)	
Greater curvature perpendicular	222 (17.9%)	21 (39.6%)	201 (16.9%)	
Middle position of gastric posterior wall	879 (70.7%)	22 (41.5%)	857 (72.0%)	
Stapler type				< 0.001
Circle stapler	879 (70.7%)	24 (45.3%)	855 (50.5%)	
Linear stapler	364 (29.3%)	29 (54.7%)	335 (49.5%)	
Position of afferent loop				0.670
Greater curvature	731 (58.8%)	33 (62.3%)	698 (58.7%)	
Lesser curvature	512 (41.2%)	20 (37.7%)	492 (41.3%)	

We summarized the univariate and multivariable logistic regression analyses (Table 2). In univariate analysis, age, BMI, preoperative pyloric obstruction, volume of residual stomach, position of anastomosis, and stapler type showed *p* values of less than 0.05. In multivariable analysis, age (OR 1.081, 95% CI, 1.047–1.117), BMI (OR 1.233, 95% CI, 1.116–1.363), preoperative pyloric obstruction (OR 3.831, 95% CI, 1.829–8.023), smaller volume of residual stomach (OR 1.838, 95% CI, 1.325–6.080), and anastomosis in greater curvature perpendicular (OR 3.385, 95% CI, 1.632–7.019) and in greater curvature parallel (OR 2.375, 95% CI, 0.963–5.861) were independent risk factors of FDGE (Table 2).

**Table 2:** Predictive factors for FDGE (1243 cases)

Predictors	*n* (%)	Univariate analysis		Multivariate Analysis	
		*P*	OR (95% CI)	*P*	OR (95% CI)
Male sex	801 (64.4%)	0.804	1.076 (0.602–1.924)		
Age, years^a^	53.444 ± 9.390	< 0.001	1.094(1.064–1.124)	< 0.001	1.081 (1.047–1.117)
BMI, kg/m^2a^	22.162 ± 3.095	< 0.001	1.229 (1.122–1.346)	< 0.001	1.233 (1.116–1.363)
Smoking history	256 (20.6%)	0.286	1.406 (0.751–2.632)		
History of abdominal surgery	160 (12.9%)	0.941	1.031 (0.457–2.326)	< 0.001	3.831 (1.829–8.023)
Preoperative pyloric obstruction	114(9.2%)	< 0.001	13.166 (7.363–23.540)		
Preoperative albumin, g/L^a^	42.022 ± 4.344	0.435	0.975 (0.916–1.038)		
Preoperative hemoglobin, g/L^a^	137.370 ± 12.745	0.541	1.007 (0.984–1.030)		
Postoperative albumin, g/L^a^	34.139 ± 3.731	0.862	1.007 (0.935–1.084)		
Postoperative hemoglobin, g/L^a^	115.930 ± 13.409	0.063	1.022 (0.999–1.045)		
Preoperative anxiety					
None	751 (60.4%)	0.958	Control		
Counseling	219 (17.6%)	0.845	1.075 (0.520–2.223)		
Drugs	273 (22.0%)	0.870	0.943 (0.469–1.899)		
Operation time, min^a^	186.138 ± 21.486	0.320	1.006 (0.994–1.019)		
Operation blood loss, ml^a^	171.440 ± 52.732	0.170	1.003 (0.999–1.008)		
Approach					
Opening	832(66.9%)	Control			
Laparoscopy	411(33.1%)	0.171	1.478 (0.845–2.587)		
Reconstruction					
Roux-en-Y	442(35.6%)	0.074	Control		
Uncut Roux-en-Y	801(64.4%)	0.022	0.604 (0.348–1.050)	< 0.027	
Volume of residual stomach					
LGPA preserved	560 (45.1%)	0.035	Control	0.170	
LGPA ligation	322 (25.9%)	0.008	2.174 (1.057–4.471)	0.007	1.763 (0.785–3.963)
First branch of SGA ligation	361 (29.0%)	< 0.001	2.531(1.278–5.014)	0.004	1.838 (1.325–6.080)
Position of anastomosis					
Greater curvature parallel	142 (11.4%)	0.006	2.951 (1.367–6.371)	0.061	2.375 (0.963–5.861)
Greater curvature perpendicular	222 (17.9%)	< 0.001	4.070 (2.195–7.546)	0.001	3.385 (1.632–7.019)
Middle position of gastric posterior wall	879 (70.7%)	Control			
Stapler type					
Circle stapler	879 (70.7%)	Control			
Linear stapler	364 (29.3%)	< 0.001	3.07 (2.334–3.806)		
Position of Anastomotic					
Greater curvature	731 (58.8%)	Control			
Lesser curvature	512 (41.2%)	0.602	0.860 (0.488–1.516)		

^a^Mean ± SD

**Fig. 1:**
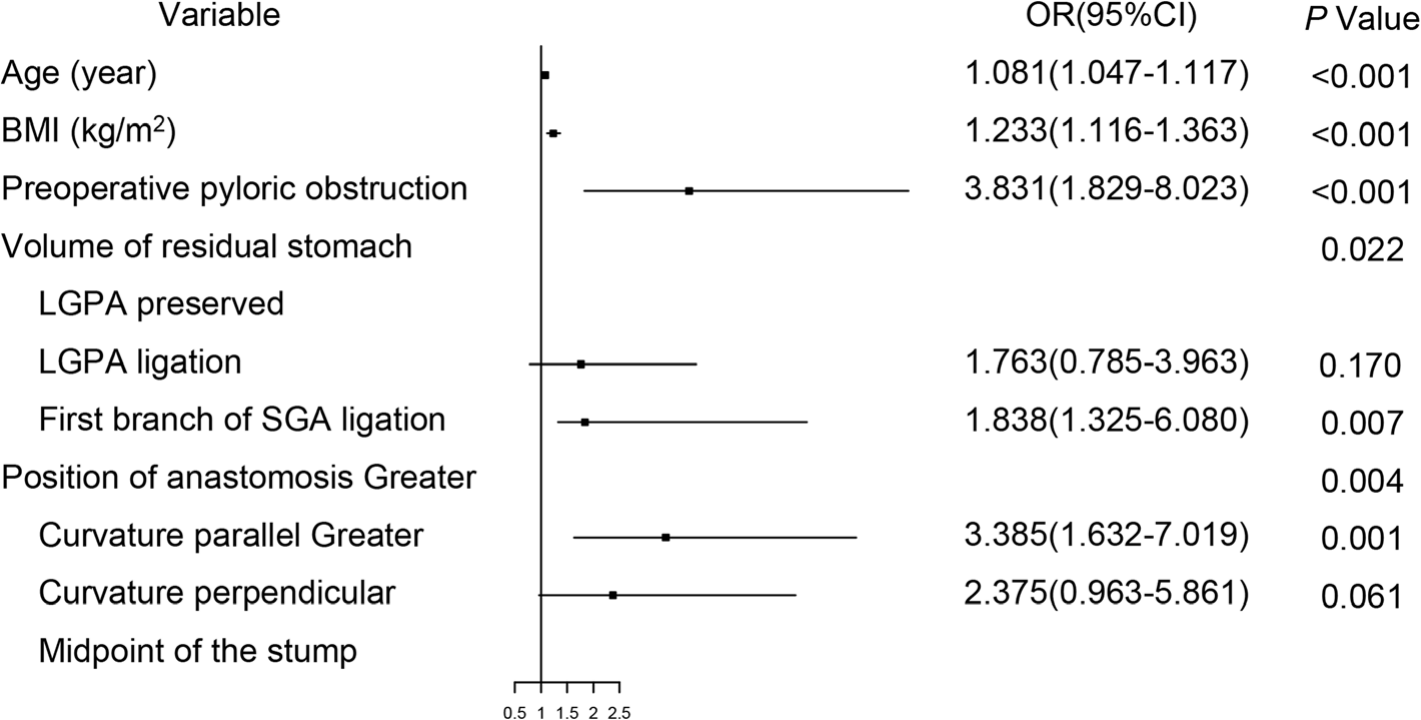
The forest plot for multivariate analysis of FDGE

Figure 1 shows that the multivariate risk analysis related to FDGE showed that preoperative obstruction, whether LGPA or SGA was ligation, greater curvature perpendicular, using linear stapler are all independent risk factors for FDGE.

In subgroup analysis of 114 patients with preoperative obstruction, clinicopathological characteristics were shown in Table 3. In univariate analysis, preoperative patients with poor nutritional status, longer preoperative obstruction, and severe obstruction were significantly associated with FDGE (Table 4). In multivariate analysis, higher BMI (OR 1.309, 95% CI, 1.086–1.579) and preoperative obstruction time (OR 1.054, 95% CI, 1.003–1.108) were independent risk factors of FDGE, and preoperative gastrointestinal decompression (OR 0.231, 95% CI, 0.068–0.785) was independent protective factor of FDGE (Table 4).

**Table 3:** General Characteristics of 114 preoperative pyloric obstruction patients

Items	Overall (*n* = 114)	FDGE (*n* = 27)	None FDGE (*n* = 87)	*P*
Male sex	88 (77.2%)	20 (74.1%)	68 (78.2%)	0.793
Age, years^a^	61.746 ± 11.835	60.111 ± 10.811	62.253 ± 12.149	0.414
BMI(kg/m^2^)^a^	22.775 ± 3.333	25.101 ± 2.689	22.053 ± 3.191	0.001
Smoking history	26 (22.8%)	8 (29.6%)	18 (20.7%)	0.431
History of abdominal surgery	17 (14.9%)	4 (14.8%)	13 (14.9%)	1.000
Preoperative albumin, g/L^a^	39.675 ± 5.012	42.296 ± 3.646	38.862 ± 5.115	0.002
Preoperative hemoglobin, g/L^a^	121.395 ± 26.799	133.296 ± 25.822	117.701 ± 26.150	0.008
Postoperative albumin, g/L^a^	32.404 ± 4.370	34.074 ± 4.367	31.885 ± 4.263	0.022
Postoperative hemoglobin, g/L^a^	113.570 ± 24.093	120.667 ± 27.354	111.368 ± 22.711	0.080
Preoperative gastric decompression, g/L^a^	66 (57.9%)	8 (29.6%)	58 (66.7%)	0.001
Periods of obstruction, days^a^	16.430 ± 10.908	20.926 ± 16.786	15.034 ± 7.926	0.014
Level of obstruction				0.025
None food retention	22 (19.3%)	10 (37.0%)	12 (13.8%)	
Small amount	60 (52.6%)	12 (44.4%)	48 (55.2%)	
Large amount	32 (28.1%)	5 (18.5%)	27 (31.0%)	
Preoperative anxiety				0.242
None	82 (71.9%)	16 (59.3%)	66 (75.9%)	
Counseling	18 (15.8%)	5 (18.5%)	9 (10.3%)	
Drugs	14 (12.3%)	6 (22.2%)	12 (13.8%)	
Operation time, min^a^	184.158 ± 49.252	195.815 ± 57.189	180.540 ± 46.2882	0.160
Operation blood loss, min^a^	193.421 ± 86.861	192.593 ± 95.780	193.678 ± 84.497	0.955
Approach				
Opening	75 (65.8%)	15 (55.6%)	60 (69.0%)	
Laparoscopy	39 (34.2%)	12 (44.4%)	27 (31.0%)	0.247
Reconstruction				
Roux-en-Y	50(35.6%)	11(40.7%)	39 (44.8%)	
Uncut Roux-en-Y	64(64.4%)	16(59.3%)	48 (55.2%)	0.825
Volume of residual stomach				
LGPA preserved	36 (31.6%)	5 (18.5%)	31 (35.6%)	
LGPA ligation	40 (35.1%)	11 (40.7%)	29 (33.3%)	0.245
First branch of SGA ligation	38 (33.3%)	11 (40.7%)	27 (31.0%)	
Position of anastomosis				
Greater curvature parallel	29 (25.4%)	7 (25.9%)	22 (25.3%)	
Greater curvature perpendicular	45 (39.5%)	13 (48.1%)	32 (36.8%)	
Middle position of gastric posterior wall	40 (35.1%)	7 (25.9%)	33 (36.8%)	0.467
Stapler type				
Circle stapler	40 (35.1%)	9 (33.3%)	31 (35.6%)	0.828
Linear stapler	74 (64.9%)	18 (66.7%)	56 (64.4%)	
Position of afferent loop				
Greater curvature	56 (49.1%)	14 (51.9%)	42 (48.3%)	
Lesser curvature	58 (50.9%)	13 (48.1%)	45 (51.7%)	0.827

^a^Mean ± SD

**Table 4:** Predictive factors for FDGE with preoperative pyloric obstruction patients (114 cases)

Predictors	*n* (%)	Univariate analysis		Multivariate analysis	
		*P*	OR (95% CI)	*P*	OR (95% CI)
Male sex	88 (77.2%)	0.659	0.798 (0.294–2.170)		
Age, years^a^	61.746 ± 11.835	0.411	0.985(0.950–1.021)		
BMI, kg/m^2a^	22.775 ± 3.333	< 0.001	1.394 (1.176–1.653)	0.005	1.309(1.086–1.579)
Smoking history	26 (22.8%)	0.336	0.620 (0.234–1.643)		
History of abdominal surgery	17 (14.9%)	0.987	1.010 (0.300–3.402)		
Preoperative albumin, g/L^a^	39.675 ± 5.012	0.003	1.175 (1.056–1.306)	0.144	1.111 (0.965–1.280)
Preoperative hemoglobin, g/L^a^	121.395 ± 26.799	0.010	1.024 (1.006–1.043)	0.601	1.006 (0.983–1.030)
Postoperative albumin, g/L^a^	32.404 ± 4.370	0.026	1.136 (1.016–1.271)	0.557	1.039 (0.915–1.178)
Postoperative hemoglobin, g/L^a^	113.570 ± 24.093	0.083	1.017 (0.998–1.036)		
Preoperative gastric decompression	66 (57.9%)	0.001	0.211(0.082–0.538)	0.019	0.231 (0.068–0.785)
Periods of obstruction	16.430 ± 10.908	0.038	1.048(1.003–1.095)	0.039	1.054 (1.003–1.108)
Level of obstruction		0.033		0.603	
None food retention	22(19.3%)	Control		Control	
Small amount	60(52.6%)	0.025	0.300(0.105–0.858)	0.348	0.523(0.135–2.027)
Large amount	32(28.1%)	0.020	0.222(0.062–0.792)	0.849	0.844(0.148–4.815)
Preoperative anxiety		0.251			
None	82 (71.9%)	Control			
Counseling	18 (15.8%)	0.184	2.292 (0.675–7.778)		
Drugs	14 (12.3%)	0.206	2.062 (0.672–6.333)		
Operation time, min^a^	184.158 ± 49.252	0.161	1.006 (0.998–1.015)		
Operation blood loss, ml^a^	193.421 ± 86.861	0.955	1.000 (0.995–1.005)		
Approach					
Opening	75 (65.8%)		Control		
Laparoscopy	39 (34.2%)	0.202	1.778 (0.734–4.306)		
Reconstruction					
Roux-en-Y	50 (35.6%)		Control		
Uncut Roux-en-Y	64 (64.4%)	0..709	1.182 (0.492–2.839)		
Volume of residual stomach		0.259			
LGPA preserved	36 (31.6%)		Control		
LGPA ligation	40 (35.1%)	0.153	2.352 (0.728–7.593)		
First branch of SGA ligation	38(33.3%)	0.123	2.526 (0.779–8.190)		
Position of anastomosis		0.472			
Greater curvature parallel	29 (25.4%)	0.500	1.500 (0.462–4.874)		
Greater curvature perpendicular	45 (39.5%)	0.221	1.915 (0.677–5.416)		
Middle position of gastric posterior wall	40 (35.1%)		Control		
Stapler type					
Circle stapler	40 (35.1%)		Control		
Linear stapler	74 (64.9%)	0.828	1.428 (1.073–1.783)		
Position of afferent loop					
Greater curvature	56 (49.1%)		Control		
Lesser curvature	58 (50.9%)	0.746	0.867 (0.365–2.057)		

^a^Mean ± SD

**Fig. 2:**
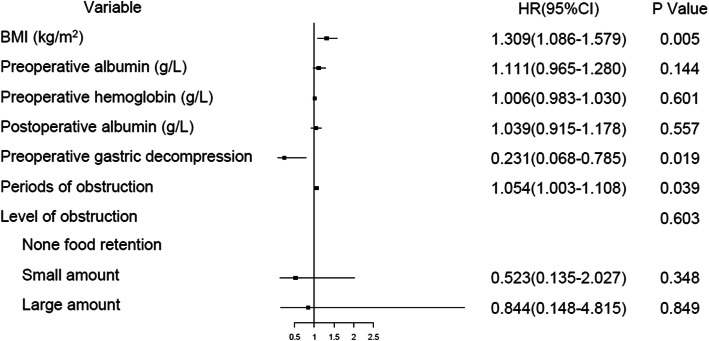
The forest plot for multivariate analysis of FDGE with preoperative pyloric obstruction patients

Figure 2 shows that the high BMI index and preoperative albumin reduction in patients with preoperative obstruction were independent risk factors for postoperative FDGE; furthermore, the use of gastrointestinal decompression before surgery can effectively relieve the occurrence of FDGE.

## Discussion

FDGE, whose main clinical manifestations includes nausea, bloating, vomiting, massive gastric juice (800 ml or more), and mechanical obstruction, is a common complication of gastrectomy [7]. The cause of the disease is still unclear, while most scholars generally believe that postoperative FDGE after gastrectomy is due to the destruction of gastric integrity, especially the vagus nerve and Cajal cells located in the middle of the stomach. Furthermore, excessive tension in the stomach tissue leads to contraction dysfunction, which causes reduction of gastric emptying[ 7]. Simre ´NM [8] found that patients with FDGE who without surgical history are related to psychological factors such as emotional stress, depression, and anxiety. Of course, these factors also play an important role in the development of FDGE. Some scholars believed that there are some genetic correlations in FDGE, such as G-protein β polypeptide-3 GNB3 genetic polymorphisms [9] and GNB3 TT genotype and CC genotype [10]. However, there was no clear study relating to surgical methods (including resection and anastomosis) and preoperative prevention.

In this study, we analyzed 53 cases of patients with FDGE after radical resection of distal gastric cancer. We analyzed correlation of preoperative conditions and surgical methods. And we found that there was the obstruction before surgery, the size of remnant stomach, the position of anastomotic stoma, and the choice of stapler were the main independent risk factors for FDGE. There was no significant correlation with age, gender, preoperative anxiety, preoperative and postoperative nutritional status, open or laparoscopic, the mode of reconstruction, operation time, and surgical bleeding volume.

Preoperative obstruction is an important factor in the occurrence of FDGE after distal gastrectomy. Some studies have shown that the factor increased the likelihood of FDGE by 26 times [11]. We performed subgroup analysis of the group of patients. It was found that gastrointestinal decompression before surgery could effectively prevent the occurrence of FDGE in these patients. There may be two reasons for this: (1) Preoperative obstruction caused gastric wall edema and gastric smooth muscle tear, resulting in gastric peristalsis weakened, nerve conduction disorders; (2) postoperative anastomotic edema and accumulation of gastrointestinal mucosa. From postoperative gastroscopy, we can also find that most patients with FDGE have different degrees of anastomotic edema.

According to the principle of surgery, we believe that during the operation, the residual gastric tissue should be retained as much as possible on the premise of ensuring the cutting edge. Meanwhile, the first branch of the short gastric blood vessels or the left gastroepiploic artery should be correspondingly retained. If the first branch of SGA is ligation, in order to ensure the blood supply and anastomosis of the remnant stomach, the surgeon will disconnect it at a higher position of the greater curvature of the stomach. Thereby, this results in the residual gastric cavity being too small, which is more likely to cause FDGE. Because Billroth I was rarely performed, our study did not involve patients undergoing Billroth I, but some studies have shown that the time of FDGE after Billroth I is longer than the Billroth II [12]. In order to achieve a better anti-reflux effect, we have now abandoned the traditional Billroth II, and the Roux-en-Y or uncut-Roux-en-Y methods were used. However, in our study, these two reconstruction methods have no significant difference in the occurrence of postoperative FDGE.

**Fig. 3:**
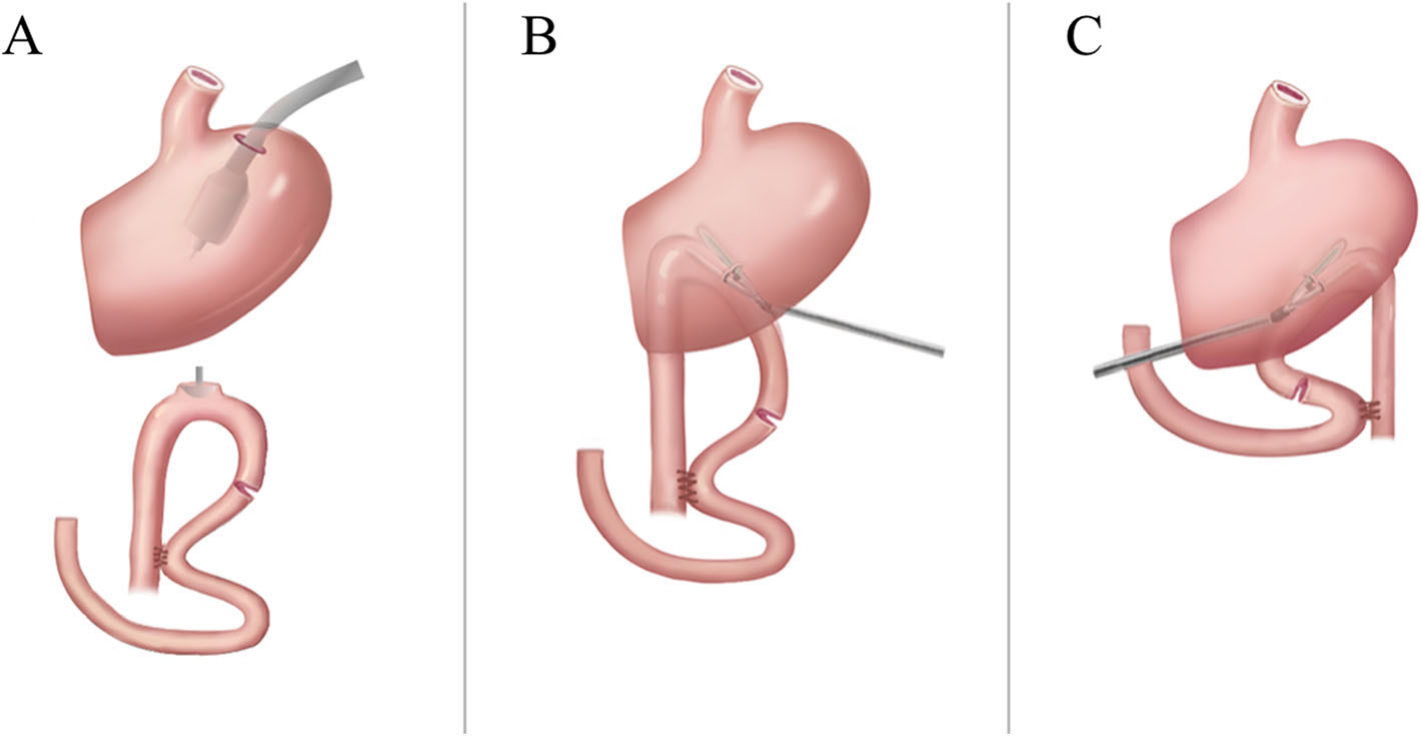
**a** Using the circular stapler to conform to the jejunum through the posterior wall of the stomach. **b** Greater curvature perpendicular of the stomach with straight linear stapler to match the posterior wall of the stomach with the jejunum. **c** Greater curvature parallel of the stomach with straight linear stapler to match the posterior wall of the stomach with the jejunum

The linear stapler is widely used in laparoscopic surgery. In 2002, the Japanese scholar Kanaya et al. [13] reported a new technique for gastroduodenal anastomosis-triangular anastomosis. He believed that this method was fit, safe, and feasible. Patients had an earlier eating time and a significantly reduced incidence of dumping syndrome [14]. Kojima et al. reported that a gastrointestinal anastomosis was performed with a linear anastomosis after distal gastrectomy in 68 patients undergoing laparoscopically assisted small incisions. Only one patient developed anastomotic stenosis and was relieved by conservative treatment. Meta-analysis results showed that the incidence of anastomotic leakage and stenosis in the straight-line anastomosis in gastric digestive tract reconstruction is significantly lower than that of circular anastomosis [15]. However, in this study, FDGE is more likely to occur in linear anastomoses than in circular anastomoses (Fig. 3a), and the parallel large curved side of the stomach (Fig. 3c) that was incised using the linear stapler is more likely to cause FDGE than the vertical large curved side of the stomach (Fig. 3b). Why did this happen? We thought anastomosis of parallel great curvature of stomach may destroy the gastric fundus pacing point, and anastomosis of vertical great curvature of stomach may result in incomplete obstruction caused by the angle formation of anastomotic outflow tract. In the gastroscopy of patients with FDGE, we often found that the anastomotic mucosa accumulates edema, and the angle formation may not be ruled out. At this time, although the gastroscope is smooth, the angulation and mucosal accumulation may hinder the emptying of the stomach contents, which may be caused by auxiliary small incision during the operation. Because of the small incision, the linear stapler cannot form the right angle of vertical gastric curvature in limited space, thus it causes the outflow track to rise. Dae Hoon Kim [6] proposed that laparoscopic distal gastrectomy is a risk factor for FDGE; this may be related to the mode of anastomosis. Another reason for FDGE might be the actual area of the anastomosis formed by the straight stapler is less than the anastomosis of the circular stapler with diameter over 25 mm, or it is more likely to cause anastomotic edema and mucosal accumulation, even the angle formation. This is consistent with the study by Kim KH [6] where he found that an anastomotic dilatation after a relatively small diameter of distal gastrectomy may be associated with a gradual improvement in FDGE. According to the above, we recommend that circular stapler be used for anastomosis. If a linear stapler is selected, it is necessary to ensure that the direction of anastomotic outflow track is downward as far as possible, so that it can naturally expand under the action of gravity.

In this study, the incidence of FDGE in laparoscopic surgery (5.4%) was higher than that in open surgery (3.7%), but there was no statistical difference. Hongbo Meng [16] also reported similar findings with open stomach surgery. The incidence of FDGE in open surgery was lower than that of laparoscopic surgery (3.7% vs. 6.9%). He thought that this may be caused by a small sample size. However, the sample size in our study is large, and this difference still exists. This may be related to the choice of the stapler in different surgical methods. More choice of circular stapler was in open surgery, while laparoscopic surgery is more to apply linear stapler. Further, whether the choice of stapler leads to surgical methods affecting the occurrence of FDGE is still to be further studied.

## Conclusion

In brief, this study found that the occurrence of FDGE is related to many factors. We can effectively prevent the occurrence of FDGE by improving preoperative obstruction, nutritional support, optimizing surgery, and anastomosis; retaining the first branch of the short gastric artery or the left gastric artery; and when performing a straight-line anastomosis, keep the outflow track in the downward direction, so that the intestine maintains natural expansion under the action of gravity. Furthermore, we should strive to explore new ideas to further reduce complications.

## Supplementary Information

**Additional file 1 **: **Supplementary Material** Flow diagram of patients enrollment and study design

## Data Availability

The datasets used and/or analyzed during the current study are available from the corresponding author on reasonable request.
