# Supplementary material for: Analysis of risk factors and prevention strategies for functional delayed gastric emptying in 1243 patients with distal gastric cancer
Source: World J Surg Oncol. 2020 Nov 19;18:302. doi: 10.1186/s12957-020-02085-2 (PMC7678317; doi:10.1186/s12957-020-02085-2)
Supplement: Supplementary file 1 — Additional file 1 : Supplementary Material Flow diagram of patients enrollment and study design [file 12957_2020_2085_MOESM1_ESM.docx]

Patients with postoperative gastroparesis (n=26)

Patients with postoperative gastroparesis (n=27)

Patients without postoperative gastroparesis (n=87)

Patients without postoperative gastroparesis (n=1103)

Patients without preoperative pyloric obstruction (n=1129)

Patients with preoperative pyloric obstruction (n=114)

Patients include in this study (n=1243)

Patients excluded (n=49)

- More than distal gastrectomy (n=17)
- Preoperative chemotherapy or irradiation (n=20)
- Diabetes mellitus (n=12)

Patients excluded (n=1452)

- Total gastrectomy (n=1135)
- Proximal gastrectomy (n=56)
- Completion gastrectomy (n=261)

GC patients in database (n=2834)

Patients include in this study (n=1382)
